# Down‐regulation of long non‐coding RNA HOTAIR inhibits invasion and migration of oesophageal cancer cells via up‐regulation of microRNA‐204

**DOI:** 10.1111/jcmm.14502

**Published:** 2019-08-07

**Authors:** Ai‐Hua Wang, Peng Tan, Yuan Zhuang, Xiu‐Tian Zhang, Zong‐Bu Yu, Lu‐Ning Li

**Affiliations:** ^1^ Department of Gastroenterology Linyi People's Hospital Linyi P.R. China; ^2^ Internal Medicine Teaching and Research Section Shandong Medical College Linyi P.R. China; ^3^ Histology and Embryology Teaching and Research Section Shandong Medical College Linyi P.R. China

**Keywords:** homeobox C8, HOX transcript antisense RNA, invasion, long non‐coding RNA, MicroRNA‐204, migration, oesophageal cancer, proliferation

## Abstract

Oesophageal cancer is a progressive tumour with high mortality. However, therapies aimed at treating oesophageal cancer remain relatively limited. Accumulating studies have highlighted long non‐coding RNA (lncRNA) HOX transcript antisense RNA (HOTAIR), microRNA‐204 (miR‐204) and homeobox C8 (HOXC8) in the progression of oesophageal cancer. Herein, we tried to demonstrate the function of HOTAIR, miR‐204 and HOXC8 in oesophageal cancer and their relationship. Differentially expressed genes involved in oesophageal cancer were identified. The endogenous expression of HOTAIR and miR‐204 in oesophageal cancer cell lines was altered to elucidate their effects and to identify the interaction among HOTAIR, miR‐204 and HOXC8. We also explored the underlying regulatory mechanisms of HOTAIR and miR‐204 with siRNA against HOTAIR, miR‐204 mimic or miR‐204 inhibitor. Cell proliferation, migration, invasion and apoptosis were subsequently detected. Xenograft in nude mice was induced to evaluate tumourigenicity. miR‐204 was down‐regulated, while HOTAIR and HOXC8 were up‐regulated in the oesophageal cancer tissues. HOTAIR could competitively bind to miR‐204 and miR‐204 could further target HOXC8. The oesophageal cancer cells treated with si‐HOTAIR or miR‐204 mimic exhibited decreased expression levels of HOXC8, Vimentin and MMP‐9, but increased E‐cadherin level. Silenced HOTAIR or elevated miR‐204 inhibited proliferation, migration and invasion, along with stimulated apoptosis of oesophageal cancer cells. In summary, our results show that lncRNA HOTAIR could specifically bind to miR‐204 as a competing endogenous RNA and regulate miR‐204 and HOXC8. Hence, down‐regulation of HOTAIR could inhibit progression of oesophageal cancer, indicating a novel target for oesophageal cancer treatment.

## 1 INTRODUCTION

Oesophageal cancer, an aggressive cancer, has been reported to be the sixth most deadly cancer globally.^1^ There are two major histological types of oesophageal cancer: squamous cell carcinoma and adenocarcinoma,^2^ and they both show a typical syndrome known as dysphagia, which negatively affect the quality of life of the patients.^3^ Oesophageal cancer is often accompanied by a high mortality mainly due to the low rate of early diagnosis, consequently resulting in a poor prognosis,^4^, ^5^ although a pre‐operative chemoradiotherapy has been reported to be able to improve the survival rate of the patients who have potentially curable oesophageal or oesophagogastric‐junction cancer.^6^ Existing literature has implicated long non‐coding RNAs (lncRNAs) in the occurrence and development of numerous aggressive tumours.^7^, ^8^ Thus, the current study aimed to investigate the role of lncRNAs might play in the progression of oesophageal cancer.

Long non‐coding RNAs are a family of RNAs that have no coding capacity however still exhibit the ability to play a crucial role in various biological regulatory processes^9^ such as a performance as a competing endogenous RNA (ceRNA) influencing post‐transcriptional regulation by interfering the pathways of microRNA (miRNA or miR).^10^ A previous study revealed the diagnostic potential of serum lncRNA HOX transcript antisense RNA (HOTAIR) as a promising biomarker for oesophageal squamous cell carcinoma.^11^ HOTAIR is a 2148‐nucleotide‐long lncRNA that has been shown to participate in the development of the physiological epidermis as well as the progression of cancer, and potentially as a regulator of tumour suppressor genes.^12^ Apart from lncRNA, accumulating evidence has highlighted the crucial inhibitory role of miRs in blocking the development of oesophageal cancer, such as microRNA‐204 (miR‐204), which performs as a tumour suppressor.^13^, ^14^, ^15^, ^16^ HOTAIR has been reported to influence the progression of oesophageal squamous cell carcinoma by binding to endogenous miR‐125 and miR‐143.^17^ Besides, studies have also suggested that HOTAIR regulates HOX genes,^18^, ^19^ whose expression has been detected in oesophageal cancer cell lines, including HOXC8.^20^ Homeobox C8 (HOXC8) is a transcription factor capable of stimulating oncogenes in various malignancies, and it is implicated in the modulation of multiple proteins that linked with cancer.^21^ Although the aforementioned literature has highlighted a relationship between HOTAIR, miR‐204 and HOXC8, their functions in the development of oesophageal cancer remain unknown. Hence, the current study aimed to investigate its underlying molecular mechanism.

## 2 MATERIALS AND METHODS

### 2.1 Ethics statement

The current study was performed with the approval of the ethics committee of The Linyi People's Hospital. All participants signed informed consents. All animal experiments were conducted in strict accordance with the Guide for the Care and Use of Laboratory Animal by International Committees.

### 2.2 Microarray‐based gene expression profiling

Gene expression data of oesophageal cancer were downloaded from the Cancer Genome Atlas (TCGA) (http://cancergenome.nih.gov/) database. Differential analysis was conducted in order to analyse the transcriptome profiling data using the R package “edgeR”^22^ False positive discovery (FDR) correction was performed based on *P*‐value with package “multitest”. FDR < 0.05 and |log2 (fold change)| >2 was set as the threshold for screening the differentially expressed genes (DEGs). The lncRNA‐binding miRNA candidates were identified from the miRcode website (http://www.mircode.org/), and the target genes of miRNAs were predicted based on the miRTarBae website (http://mirtarbase.mbc.nctu.edu.tw/ php/index.php).

### 2.3 Study subjects

Forty‐six patients (24 males and 22 females ranging from 44 to 72 years old with the mean age of 58.33 ± 9.13 years) who had been diagnosed with oesophageal cancer and underwent surgical resection at the Linyi People's Hospital from 1 July 2015 to 30 December 2016 were enrolled for this study. None of the participants received radiotherapy or chemotherapy prior to this study. Among the 46 patients, 7 patients had well‐differentiated squamous carcinoma, 15 patients had moderately differentiated squamous cell carcinoma and 24 patients had poorly differentiated squamous cell carcinoma. According to the oesophageal cancer staging criteria developed by the Union for International Cancer Control (UICC),^23^ 18 patients were at the T1 or T2 stage, and 28 patients were at the T3 or T4 stage. 31 patients had lymph node metastasis (LNM) while the remaining did not. Meanwhile, adjacent normal tissues (normal mucous tissues ≥10 cm from oesophageal cancer tissues) from all enrolled participants were obtained immediately following surgery and regarded as the control group. The collected tissues were stored at −80°C prior to use.

### 2.4 Immunohistochemistry

The collected oesophageal cancer tissues were fixed in 4% paraformaldehyde for 24 hours, dehydrated by 80%, 90% and 100% ethanol and n‐butyl alcohol, then immersed in a 60°C wax box, embedded and sliced into 5 μm sections for immunohistochemistry assay. The sections were subsequently baked at 60°C for 1 hour and then dewaxed by xylene, followed by dehydration with gradient alcohol, immersion in 3% H_2_O_2_ for 10 minutes and wash with distilled water. After high‐pressure antigen retrieval for 90 seconds, the sections were cooled to room temperature and washed three times with phosphate buffer saline (PBS), 3 minutes per wash. The slices were then blocked with 100 μL 5% bovine serum albumin (BSA) at 37°C for 30 minutes. Diluted primary rabbit antibody to HOXC8 (100 μL, 5 μg/mL, ab86236, Abcam Inc, Cambridge, MA) was added to the slices and incubated at 4°C overnight. After three PBS washes, the tissue samples were incubated with biotin‐labelled goat anti‐rabbit secondary antibody (1:100, HY90046, Shanghai Hengyuan Biotechnology, Shanghai, China) working solution at 37°C for 30 minutes. After an additional PBS wash, streptomycin anti‐biotin peroxidase solution (Beijing Zhongshan Biotechnology, Beijing, China) was added to the slices, followed by prompt incubation at 37°C for 30 minutes. After three PBS washes, diaminobenzidine (DAB) (Beijing Bioss, Beijing, China) was employed for tissue colouration at room temperature, after which the sections were immersed in haematoxylin for 5 minutes, washed with running water, rinsed in 1% hydrochloric ethanol for 4 seconds and treated under running water for 20 minutes to visualize a blue colour. The Image‐Proplus image analysis software (Media Cybernetics, Silver Springs, MD) was employed to determine the average optical density (OD) value of HOXC8 positive staining, with brown‐yellow coloured cells observed^24^ at high magnification. Five fields of vision (×400) were randomly selected from each section with 200 cells from each field, with the percentage of positive cells calculated accordingly. Each experiment was repeated independently for three times, after which the mean value was obtained.

### 2.5 Cell line selection

Five oesophageal cancer cell lines, namely EC8712, EC8733, ECA109, EC9706 and EC8501 as well as normal oesophageal epithelial cell line HEEC (Shanghai Institute of Biochemistry and Cell Biology, Shanghai, China) were cultured in the RPMI 1640 medium containing 10% serum at 37°C with 5% CO_2_. The medium was replaced every 2‐3 days based on cell growth. The cells were passaged when they covered 80%‐90% of the culture plates. Reverse transcription quantitative polymerase chain reaction (RT‐qPCR) was performed in order to select the cell lines with the highest HOTAIR and miR‐204 expression for further experimentation.

### 2.6 Dual luciferase reporter gene assay

The interaction site between miR‐204 and HOTAIR or HOXC8 was predicted by the biological prediction website (https://cm.jefferson.edu/rna22/) and the binding sequences were obtained to construct luciferase‐tag plasmids. The full length of HOTAIR and the 3'untranslated region (3'UTR) of HOXC8 were transferred, respectively, to the pmirGLO luciferase vector (E1330, Promega Corporation, Madison, WI) via clonal amplification and referred to as pHOTAIR‐Wt and pHOXC8‐Wt. The mutations were introduced on the binding site based on the sequence of pHOTAIR‐Wt and pHOXC8‐Wt with the resulting constructs referred to as pHOTAIR‐Mut and pHOXC8‐Mut, respectively. The pRL‐TK vector (E2241, Promega Corporation, Madison, WI) expressing renilla luciferase was considered as the internal control. Next, miR‐204 mimic and miR‐204 negative control (NC) were respectively co‐transfected with luciferase reporter vector into HEK‐293T, after which luminance was detected using a luminometer (Glomax20/20, Promega Corporation, Madison, WI).

### 2.7 RNA pull‐down assay

The cells were transfected with 50 nmol/L biotin‐labelled WT‐bio‐miR‐204 and MUT‐bio‐miR‐204. At 48 hours post‐transfection, the cells were collected, washed with PBS and then incubated in the specific lysate buffer (Ambion, Austin, Texas) for 10 minutes. The lysates were further incubated with M‐280 streptavidin beads (S3762, Sigma‐Aldrich St. Louis, MO) that had been pre‐coated with RNase‐free BSA and yeast tRNA (TRNABAK‐RO, Sigma‐Aldrich St. Louis, MO). Following a 3 hours period of incubation at 4°C, the beads were washed twice with pre‐cooled lysate buffer, thrice with low‐salt buffer and once with high‐salt buffer. The combined RNA was purified using Trizol, after which HOTAIR was detected by RT‐qPCR.

### 2.8 RNA‐immunoprecipitation assay

The cells were treated with lysis buffer (25 mmol/L Tris‐HCl pH = 7.4, 150 mmol/L NaCl, 0.5% NP‐40, 2 mmol/L ethylenediamine tetraacetic acid (EDTA), 1 mmol/L NaF and 0.5 mmol/L dithiothreitol) containing RNasin (Takara, Dalian, Liaoning, China) and protease inhibitors (B14001a, Roche, Basel, Switzerland). The lysate was centrifuged at 12 000 *g* for 30 minutes to collect supernatant. The supernatant was subsequently incubated with anti‐Ago‐2‐coated beads (BMFA‐1, BioMarker Technologies, Beijing, China) with the supernatant in the negative control incubated with anti‐immunoglobulin G (IgG)‐coated beads. After 4‐h of incubation at 4°C, washing buffer (50 mmol/L Tris‐HCl, 300 mmol/L NaCl pH = 7.4, 1 mmol/L MgCl_2_, 0.1% NP‐40) was used to wash the beads three times. The Trizol method was performed to obtain RNA from the beads, after which the expression of HOTAIR was determined by RT‐qPCR.

### 2.9 Fluorescent in situ hybridization

A Fluorescent in situ hybridization (FISH) kit (C10910, Guangzhou RiboBio Co., Ltd., Guangzhou, China) was utilized to determine the expression of HOTAIR in cells in situ. The cells exhibiting logarithmic growth were selected, detached and placed on the slides (approximately 6 × 10^4^ cells/well) of a 24‐well plate. When cell confluence had reached 60%‐70%, the cells were collected, washed with PBS for 5 minutes and then fixed with 4% paraformaldehyde at room temperature for 10 minutes, followed by three PBS washes (5 minutes per wash). The cells were then incubated with 1 mL pre‐cooled permeable fluid at 4°C for 5 minutes and then washed three times with PBS (5 minutes per wash) after the permeable fluid had been removed. The cells were subsequently blocked with 200 μL pre‐heated prehybridization solution for 30 minutes at 37°C. Hybridization solution was prepared by adding 2.5 μL 20 μmol/L FISH Probe Mix stored solution under conditions void of light. The cells were then incubated with hybridization solution containing probes overnight at 37°C under conditions void of light, after the prehybridization solution had been removed. The next day, the cells were washed three times with cleaning Lotion I (5 minutes per wash) in order to reduce the background signal, followed by washing with lotion II and once more with lotion III at 42°C under conditions void of light. Next, 4',6‐diamidino‐2‐phenylindole (DAPI) was employed to stain the cells for 10 minutes, which followed by three PBS washes at room temperature. The coverslips with migrated cells were subsequently carefully removed from the wells under dark conditions, fixed and then mounted with a medium for fluorescence detection. HOTAIR specific probe was synthesized by Ribo Biotech Co., Ltd., (Guangzhou, Guangdong, China).

### 2.10 Cell treatment

Cell lines exhibiting the highest HOTAIR expression were randomly assigned into four groups, namely: the control group (cells without any treatment), NC group (cells transfected with empty vector), si‐HOTAIR group (cells transfected with si‐HOTAIR) and HOTAIR group (cells transfected with overexpressed HOTAIR plasmid). Cell lines displaying the highest miR‐204 expression were randomly assigned into six groups, namely, the blank group (cells without any treatment), NC group (cells transfected with empty vector), HOTAIR group (cells transfected with overexpressed HOTAIR plasmid), si‐HOTAIR group (cells transfected with si‐HOTAIR), miR‐204 mimic group (cells transfected with miR‐204 mimic), miR‐204 inhibitor group (cells transfected with miR‐204 inhibitor) and si‐HOTAIR + miR‐204 mimic group (cells co‐transfected with si‐HOTAIR and miR‐204 mimic). si‐HOTAIR, miR‐204 mimic and miR‐204 inhibitor were all purchased from Ribo Biotech (Guangzhou, Guangdong, China). The cell transfection procedures were performed as follows: the cells were inoculated in a 50 mL culture bottle with complete medium until the cell density reached 50%‐60%. Next, 5 µL Lipofectamine 2000 (Gibco BRL, Grand Island, NY) was diluted with 100 µL serum‐free culture medium and the diluted mixture was permitted to stand at room temperature for 5 minutes; meanwhile, RNA (50 nmoL) or DNA (2 μg) was diluted in 100 µL serum‐free medium at room temperature for 5 minutes. Lipofectamine mixture and diluted RNA or DNA mixture were mixed and incubated for 20 minutes at room temperature in order to produce the complex of RNA/DNA with liposome. The cells were washed with serum‐free medium and then incubated with the complex for 6‐8 hours at 37°C with 5% CO_2_, after which the medium was replaced with a complete culture medium.

### 2.11 RT‐qPCR

Cells at the logarithmic growth phase were collected for total RNA extraction. The RNA concentration and purity were assessed using a ultraviolet spectrophotometer. The cDNA template was synthesized by RT reaction in the PCR amplification instrument in accordance with the instructions of the EasyScript First‐Strand cDNA Synthesis SuperMix (AE301‐02, Transgen Biotech, Beijing, China). An EP tube was subsequently added with 5 μL Mix reagent, 5 μL total RNA, 1 μL random primer and 9 μL RNase Free H_2_O, with the total subsequently mixed by centrifugation and placed in a PCR instrument (9700, Beijing Dingguo Changsheng Biotechnology, Beijing, China) for RT. The primers of U6, glyceraldehyde‐3‐phosphate dehydrogenase (GAPDH), HOTAIR, miR‐204, HOXC8, E‐cadherin, Vimentin and matrix metalloproteinase‐9 (MMP‐9) were designed and synthesized by Sangon Biotech (Shanghai) Co., Ltd., (Shanghai, China) (Table 1). The cDNA was subjected to fluorescence qPCR according to the instructions of the SYBR^®^Premix Ex TaqTM II kit (TaKaRa, Dalian, Liaoning, China). ABI7500 fluorescence qPCR (ABI Company, Oyster Bay, NY) was employed in order to perform RT‐qPCR with GAPDH and U6 regarded as the internal controls. The expression pattern of HOTAIR, miR‐204, HOXC8, E‐cadherin, Vimentin and MMP‐9 was subsequently determined. Each experiment was repeated independently three times.

**Table 1:** List of primers for RT‐qPCR

Gene	Sequences
U6	F: 5'‐GCGCGTCGTGAAGCGTTC‐3'
	R: 5'‐GTGCAGGGTCCGAGGT‐3'
GAPDH	F: 5'‐GAGAGACCCCACTTGCTGCCA‐3'
	R: 5'‐CTCACACTGCCCCTCCCTGGT‐3'
HOTAIR	F: 5'‐GACACCACTGGAGGGTGACT‐3'
	R: 5'‐CAGGTCCACATGGTCTTCCT‐3'
miR‐204	F: 5'‐CGGCGTTTGTCATCCTATG‐3'
	R: 5'‐GTGCAGGGTCCGAGGT‐3'
HOXC8	F: 5'‐ACCGGCCTATTACGACTGC‐3'
	R: 5'‐TGCTGGTAGCCTGAGTTGGA‐3'
E‐cadherin	F: 5'‐CGAGAGCTACACGTTCACGG‐3'
	R: 5'‐GGGTGTCGAGGGAAAAATAGG‐3'
Vimentin	F: 5'‐GACGCCATCAACACCGAGTT‐3'
	R: 5'‐CTTTGTCGTTGGTTAGCTGGT‐3'
MMP‐9	F: 5'‐TGTACCGCTATGGTTACACTCG‐3'
	R: 5'‐GGCAGGGACAGTTGCTTCT‐3'

Abbreviations: F, forward; GAPDH, glyceraldehyde phosphate dehydrogenase; HOXC8, homeobox C8; miR‐204, microRNA‐204; MMP‐9, matrix metalloproteinase‐9; R, reverse; RT‐qPCR, reverse transcription quantitative polymerase chain reaction.

### 2.12 Western blot analysis

The cells were initially washed three times with pre‐cooled PBS, following a 48 hours period of transfection. After additional PBS washing, the cells were incubated with radioimmunoprecipitation assay (RIPA) lysis buffer (Beyotime Biotechnology Co., Shanghai, China) in a 1.5 mL centrifugation tube. After, centrifugation at 14 000 *g* for 10 minutes and collection of the supernatant, the protein concentration was determined using the bicinchoninic acid (BCA) method, after which the supernatant was stored at −20°C. The collected proteins were subjected to electrophoresis separation using 10% separation gel and 5% spacer gel that were prepared using a sodium dodecyl sulfate‐polyacrylamide gel electrophoresis kit. The separated proteins were transferred to the nitrocellulose membrane via the wet method, and blocked with 5% BSA at room temperature for 1 hour. The membrane was then incubated with diluted primary antibodies to HOXC8 (ab79690, 1:200), E‐cadherin (ab1416, 1:50), Vimentin (ab8978, 1:100), MMP‐9 (ab73734, 1:500), and GAPDH (ab37168, 1:200) at 4°C overnight. All above antibodies were purchased from Abcam Inc, (Cambridge, MA). On the next day, the membrane was washed three times with phosphate buffered saline tween‐20 (PBST) (10 minutes each time) and then incubated with diluted (in 5% skimmed milk) secondary antibody of goat anti‐rabbit polyclonal antibody (ab7312, Abcam Inc, Cambridge, MA) on a shaking table at room temperature for 1 hour. After washing the membrane with PBST for three times (15 minutes per wash), the sample was subjected to film development using the developer and the Bio‐Rad gel imaging system (MG8600, Thmorgan, Beijing, China). The IPP 7.0 software (Media Cybernetics, Singapore) which was used for quantitative analyses.

### 2.13 3‐(4,5‐dimethyl‐2‐thiazolyl)‐2,5‐diphenyl‐2‐H‐tetrazolium bromide (MTT) assay

After a 48 hours period of transfection, cells at the logarithmic growth phase were harvested and then resuspended in a RPMI 1640 culture medium containing 16% foetal bovine serum (FBS) into a density of 2.5 × 10^5^ cells/mL. Next, the cell suspension was then transferred into a 96‐well plate (8 wells were set in each group; 100 μL/well) and cultured at 37°C with 5% CO_2_. The plate was then removed after 24, 48, 72 hours of incubation, respectively, with 10 μL 5 mg/mL MTT solution added to each well (Sigma‐Aldrich, St. Louis, MO). After an additional 4‐h period of incubation, the cells were collected with the supernatant removed accordingly. Next, 150 μL of dimethylsulphoxide was added to each well and gently shaken for 10 minutes in order to elicit adequate dissolution. Finally, the OD values at 490 nm were measured using a microplate reader (Bio‐Rad Laboratories, Hercules, CA). Each experiment was repeated independently for three times.

### 2.14 Scratch test

Following 48 hours of transfection, the transfected cells in each group were seeded in a 6‐well plate (5 × 10^5^ cells/well). In the event of cell confluence reaching approximately 90%, a scratch was made using a sterile pipette along the central axis across the well. Cells failing to adhere to the wall were removed by PBS washing, with serum‐free culture medium subsequently added for an additional 0.5‐1‐h culture to induce cell recovery. The cells were photographed at 0 and 24 hours after cell recovery. The Image‐Pro Plus Analysis software (Version X; Media Cybernetics, Silver Springs, MD) was employed to measure cell migration distance. The relative migration rate was calculated with the blank group regarded as the control based on the following formula: relative migration rate = (migration distance_experimental group_/migration distance_blank group_) × 100% (the control value was 1 or 100%). The experiment was independently repeated three times.

### 2.15 Transwell assay

The cells that had undergone a 48 hours period of transfection were dissolved in Matrigel (356234, BD Biosciences, San Jose, CA) at 4°C overnight, which was then diluted in serum‐free medium (1:3), added to the apical chamber of a Transwell chamber (50 μL/well) and then incubated for 30 minutes. After incubation, the cells were detached, washed three times with serum‐free culture medium, tallied and resuspended accordingly. Matrigel was washed with serum‐free medium, after which the cell suspension was inoculated into the apical chamber (1 × 10^5^ cells/mL). The serum‐free medium was added to the apical chamber with the medium containing 10% FBS added to the basolateral chamber. After incubation for 24 hours at 37°C, the Transwell chamber was rinsed twice with PBS (5 min per time), fixed by 5% glutaraldehyde at 4°C and stained with 0.1% crystal violet for 30 minutes. The Transwell chamber was washed twice with PBS and then observed under a microscope. The number of cells invading the Matrigel was considered to be a reflection of the invasion ability. Each experiment was independently repeated three times.

### 2.16 Flow cytometry

Annexin V‐fluorescein isothiocyanate (FITC)/propidium iodide (PI) double staining kit (556547, Shanghai Shuojia Biotechnology Co. Ltd., Shanghai, China) was employed to detect cell apoptosis after 48‐h transfection. Prior to detection, the cells were diluted using deionized water. After centrifugation at 716 *g* for 5 minutes, the cells in each group were collected, followed by cell re‐suspension. After additional centrifugation for 5‐10 minutes, the cells were washed and resuspended with 300 μL 1 × binding buffer. Next, 5 µL Annexin V‐FITC was added to the cells, mixed entirely and incubated at room temperature for 15 minutes under dark conditions. Five minutes prior to the use of a flow cytometer (Cube6, Partec, Germany), 5 µL PI was added to the cells, which were incubated in an ice bath under dark conditions. FITC was detected at an excitation wavelength of 488 nm and 530 nm while PI at a wavelength >575 nm.

### 2.17 Tumour xenografts in nude mice

A total of 66 clean Kunming nude mice (age: 4‐6 weeks, weight: 16‐22 g) purchased from Animal Experiment Center, Southern Medical University (Guangzhou, Guangdong, China) were recruited for this study. The selected cell lines were utilized to prepare a single cell suspension in PBS and Matrigel (1:1) mixture (the final concentration of the cells were diluted to 1 × 10^6^ cells/200 μL). The nude mice were evenly divided into 11 groups, anaesthetized with ether and then injected subcutaneously into the back of the right hind leg with the cells (1 × 10^6^ cells/200 μL) that had been transfected with different plasmids. After cell injection, the mice were fed under controlled conditions and analysed every 7 days. The length and width of the tumours were recorded with the tumour volume calculated according to the following formula: tumour volume = length × width^2^/2.

### 2.18 Statistical analysis

All experimental data were analysed using SPSS 21.0 statistical software (IBM Corp., Armonk, NY). Measurement data were expressed as mean ± SD. Kolmogorov‐Smirnov was employed to assess normal distribution. Data of oesophageal cancer tissues and adjacent normal tissues that conformed to normal distribution were analysed by paired *t* test and those that conformed to skewed distribution were analysed by the non‐parametric Wilcoxon signed‐ranks test. Data from multiple groups were compared by one‐way ANOVA. Pairwise comparisons between mean values were analysed by least significant difference (LSD) and cell viability or tumour volume at different time‐points was compared by repeated measurement ANOVA. A *P* < 0.05 was considered to be statistically significant.

## 3 RESULTS

### 3.1 miR‐204 is down‐regulated while HOTAIR and HOXC8 are up‐regulated in oesophageal cancer tissues

**Figure 1:**
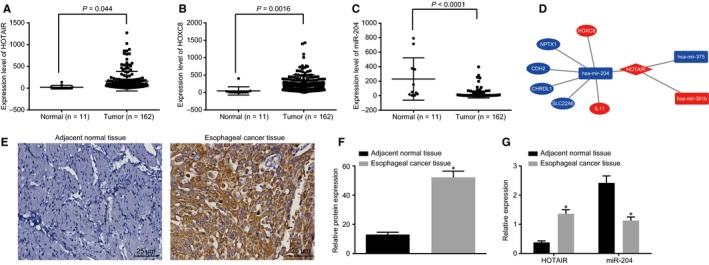
microRNA‐204 (miR‐204) has low expression level while HOTAIR and HOXC8 have high expression level in oesophageal cancer tissues. (A), HOTAIR is highly expressed in oesophageal cancer tissues based on the data from TCGA database; (B) HOXC8 is highly expressed in oesophageal cancer tissues based on the data from TCGA database; (C) miR‐204 is lowly expressed in oesophageal cancer tissues based on the data from TCGA database; (D) possible network of HOTAIR in oesophageal cancer as a ceRNA. (E,F), immunohistochemistry reveals that the positive expression rate of HOXC8 is significantly elevated in oesophageal cancer tissues (400x); (G) RT‐qPCR confirmed that HOTAIR is highly expressed while the miR‐204 is lowly expressed in oesophageal cancer tissues. The data are analysed by paired *t* test; n = 46. The experiment was independently repeated three times; *P < 0.05 vs the adjacent normal tissues

Gene expression microarray analysis was performed to screen differentially expressed lncRNAs, miRNAs and genes associated with oesophageal cancer. The TCGA database revealed that the expression of HOTAIR and HOXC8 were up‐regulated in the oesophageal cancer tissues while the expression of miR‐204 was down‐regulated (Figure 1A‐C). Analysis of the miRcode and miRTarBae websites revealed that HOTAIR could potentially interact with miR‐204 and consequently regulate the expression of HOXC8 and interleukin‐11 (IL‐11) (Figure 1D). Considering the mechanism by which IL‐11 influences cancer has been thoroughly studied,^25^, ^26^ we aimed to investigate the mechanism by which HOTAIR acting as a ceRNA of miR‐204 regulates the expression of HOXC8 in oesophageal cancer cells. Immunohistochemistry and RT‐qPCR were conducted in order to determine the expression pattern of HOXC8, HOTAIR and miR‐204 in the oesophageal cancer tissues as well as the adjacent normal tissues. The immunohistochemistry (Figure 1E,F) and RT‐qPCR (Figure 1G) results demonstrated that compared with the adjacent normal tissues, the positive expression rate of HOXC8 as well as the expression of HOTAIR was considerably elevated in the oesophageal cancer tissues, while the expression of miR‐204 was notably decreased (all *P* < 0.05). The correlation between HOTAIR, gender, age, tumour size, tumour staging, LNM, differentiation degree and invasion of fibrous membrane were analysed (Table 2), the results of which revealed that the expression of HOTAIR had no correlation with age and tumour size but was associated with tumour staging, LNM, differentiation degree and invasion of fibrous membrane. The aforementioned results implicated miR‐204, HOTAIR and HOXC8 in the development of oesophageal cancer.

**Table 2:** The expression of HOTAIR has no correlation with age and tumour size but is associated with tumour staging, LNM, differentiation degree and invasion of fibrous membrane

Baseline characteristics	HOTAIR			*P*
	Negative	Positive		
Gender				
Male	10 (41.67%)	14 (58.33%)		0.382
Female	12 (54.55%)	10 (45.45%)		
Age (y)				
<50	4 (36.36%)	7 (63.64%)	0.383	
≥50	18 (51.43%)	17 (48.57%)		
Tumour size (cm)				
<2	8 (70.73%)	3(27.27%)		0.058
≥2	14 (40.00%)	21(60.00%)		
Staging				
T1‐T2	14 (77.78%)	4 (22.22%)		0.001
T3‐T4	8 (28.57%)	20 (71.43%)		
LNM				
No	14 (93.33%)	1(6.67%)		<0.001
Yes	8 (25.81%)	23 (74.19%)		
Differentiation degree				
High	6 (85.71%)	1 (14.29%)		0.014
Moderate	8 (55.33%)	7 (46.67%)		
Poor	8 (33.33%)	16 (66.67%)		
Invasion of fibrous membrane				
Yes	8 (29.63%)	19 (70.37%)		0.003
No	14 (73.68%)	5 (26.32%)		

Abbreviation: LNM, lymph node metastasis.

### 3.2 miR‐204 binds to HOTAIR and HOXC8

**Figure 2:**
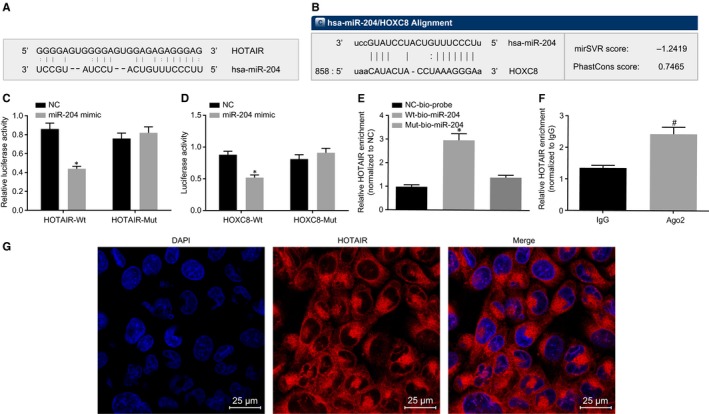
HOTAIR could bind to microRNA‐204 (miR‐204) and HOXC8 is the target gene of miR‐204. (A and C) miR‐204 binds to the HOTAIR predicted using the target prediction program and verified by the determination of luciferase activity; (B and D) miR‐204 binds to the 3'UTR of HOXC8 predicted using the target prediction program and verified by the determination of luciferase activity; (E) the RNA pull‐down assay demonstrates miR‐204 could directly bind to HOXC8; (F) the RIP assay indicates that HOXC8 could directly bind to Ago2 protein; (G) HOTAIR is mainly expressed in the cytoplasm (×400); the data are presented as mean ± SD, and analysed by one‐way ANOVA among multiple groups; pairwise comparison was conducted by paired *t* test. N = 3. The experiment was independently repeated three times. *P < 0.05 vs the NC group. ^#^ *P* < 0.05 vs the Ago2 group

Data provided by online software, suggested the existence of binding sites between miR‐204 and HOTAIR as well as between miR‐204 and HOXC8, indicating that miR‐204 could bind to HOTAIR and HOXC8. Luciferase activity determination further verified the interaction between miR‐204 and HOTAIR or HOXC8. Compared with the NC group, the luciferase activity of WT‐HOTAIR was found to be inhibited by miR‐204 (*P* < 0.05), while the luciferase activity of the MUT‐HOTAIR was unencumbered, suggesting that miR‐204 could specifically bind to HOTAIR (Figure 2A,C). A similar observation was also identified in the interaction between miR‐204 and 3'UTR of WT‐HOXC8 (*P* < 0.05), with the luciferase activity of the MUT‐HOXC8 observed to be unencumbered by miR‐204, indicating that miR‐204 could also bind specifically to HOXC8 (Figure 2B,D). RNA pull‐down as well as RIP assays was performed in an attempt to further verify the interaction among HOTAIR, miR‐204 and HOXC8. The RNA pull‐down assay revealed that the relative HOTAIR enrichment was significantly increased in WT‐miR‐204 when compared with MUT‐miR‐204 (*P* < 0.05), indicating that miR‐204 could directly bind to HOTAIR (Figure 2E). The results of the Ago2 RIP assay, revealed that compared with IgG, HOTAIR enrichment was significantly increased in Ago2 (*P* < 0.05), suggesting that HOTAIR could directly bind to Ago2 protein (Figure 2F). The aforementioned results indicated that HOTAIR could participate in the regulation of HOXC8 by competitively binding to miR‐204. The FISH results exhibited that HOTAIR was predominately distributed in the cytoplasm (Figure 2G).

### 3.3 EC9706 and ECA109 cell lines were selected for experiments

**Figure 3:**
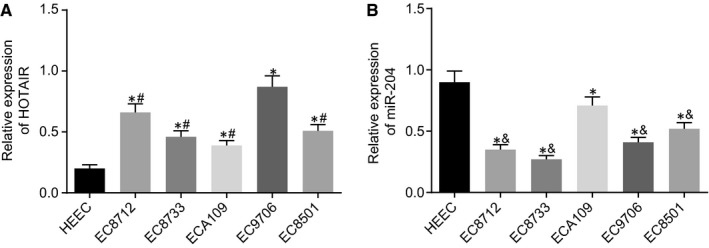
EC9706 and ECA109 cell lines exhibit highest expression of HOTAIR (A) and miR‐204 (B), respectively. *P < 0.05 vs the HEEC cell line. ^#^ *P* < 0.05 vs the EC9706 cell line. ^&^ *P* < 0.05 vs the ECA109 cell line. n = 3. The measurement data were expressed by mean ± SD and analysed by one‐way ANOVA. The experiment was independently repeated three times

Several cell lines were evaluated in order to select the ones exhibiting the highest HOTAIR and miR‐204 expression for further study. The RT‐qPCR results (Figure 3) revealed that compared with the HEEC cell line, the expression of miR‐204 was significantly lower while the expression of HOTAIR was higher in the EC8712, EC8733, ECA109, EC9706 and EC8501 cell lines (all *P* < 0.05). In comparison to the EC9706 cell line, the expression of HOTAIR exhibited an reduction in the other cell lines (all *P* < 0.05). In comparison to the ECA109 cell line, the expression of miR‐204 markedly decreased to different degrees in the EC8712, EC8733, EC9706 and EC8501 cell lines (all *P* < 0.05). Thus, based on our results, the EC9706 and ECA109 cell lines were selected for subsequent experimentation.

### 3.4 Down‐regulation of HOTAIR decreases the expression of HOXC8 and alters the expression of proteins related to cell proliferation, migration and invasion through up‐regulation of miR‐204

**Figure 4:**
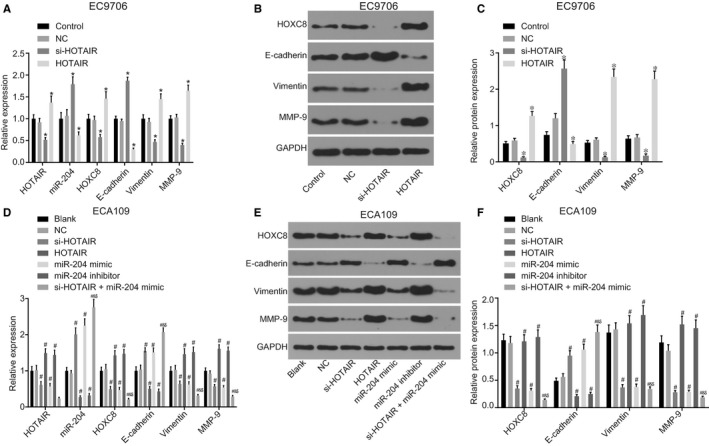
Down‐regulation of HOTAIR decreases the expression of HOXC8 and alters the protein expression levels related to cell proliferation, migration and invasion through up‐regulation of miR‐204. (A‐C), the expression of HOTAIR and miR‐204, and mRNA and protein expression of HOXC8, E‐cadherin, MMP‐9 and Vimentin in the EC9706 cell line; (D‐F), expression of HOTAIR and miR‐204 and mRNA and protein expression of HOXC8, E‐cadherin, MMP‐9 and Vimentin in ECA109 cell line; *P < 0.05 vs the control group. ^#^ *P* < 0.05 vs the blank group. ^&^ *P* < 0.05 vs the si‐HOTAIR group. ^$^ *P* < 0.05 vs the miR‐204 mimic group. The data are presented as mean ± SD, and analysed by one‐way ANOVA. n = 3. The experiment was independently repeated three times

In order to ascertain as to whether HOTAIR or miR‐204 could influence the expression of HOXC8 and proteins related to cell proliferation, migration and invasion, the relative expression levels of HOXC8, Vimentin, MMP‐9 and E‐cadherin were determined by RT‐qPCR and western blot analysis means. As illustrated in Figure 4, in the EC9706 cell line, the mRNA and protein expression levels of HOXC8, E‐cadherin, Vimentin and MMP‐9 exhibited no significant difference between the control group and the NC group (all *P* > 0.05). When EC9706 cells were treated with the si‐HOTAIR, not only did the expression of HOTAIR notably decrease but the mRNA and protein expression levels of HOXC8, Vimentin and MMP‐9 were also decreased, while the expression of miR‐204 and the mRNA and protein expression levels of E‐cadherin were remarkably increased when compared with the control group (all *P* < 0.05). When the EC9706 cells had been transfected with HOTAIR overexpression vectors (HOTAIR group), the expression of HOTAIR and the mRNA and protein expression levels of HOXC8, Vimentin and MMP‐9 were elevated while that of miR‐204 and the mRNA and protein expression levels of E‐cadherin were considerably reduced when compared to the control group (all *P* < 0.05).

In relation to the ECA109 cell line, no significant difference was detected regarding the mRNA and protein expression levels of HOXC8, E‐cadherin, Vimentin and MMP‐9 between the blank and the NC groups (all *P* > 0.05). In comparison to the blank group, the expression of HOTAIR and the mRNA and protein expression levels of HOXC8, Vimentin and MMP‐9 exhibited notable decreases in the si‐HOTAIR and si‐HOTAIR + miR‐204 mimic groups while the expression of miR‐204 and the mRNA and protein expression levels of E‐cadherin were significantly increased (all *P* < 0.05). In the miR‐204 mimic group, the mRNA and protein expression levels of HOTAIR, HOXC8, Vimentin and MMP‐9 were considerably decreased, whereas the expression of miR‐204 and the mRNA and protein expression levels of E‐cadherin were significantly increased (all *P* < 0.05). When the expression of miR‐204 was suppressed (miR‐204 inhibitor group) or the expression of HOTAIR was up‐regulated (the HOTAIR group), the expression of HOTAIR, mRNA and protein expression levels of HOXC8, Vimentin and MMP‐9 significantly increased whereas the expression of miR‐204 and the mRNA and protein expression levels of E‐cadherin were markedly decreased (all *P* < 0.05). In comparison to the si‐HOTAIR and miR‐204 mimic groups, the mRNA and protein expression levels of HOXC8, Vimentin and MMP‐9 decreased in the si‐HOTAIR + miR‐204 mimic group while the expression of miR‐204 and the mRNA and protein expression levels of E‐cadherin were notably elevated (all *P* < 0.05). The aforementioned results provided evidence indicating that the down‐regulation of HOTAIR decreases the expression of HOXC8 and alters the protein levels associated with cell proliferation, migration and invasion by up‐regulating miR‐204.

### 3.5 Down‐regulation of HOTAIR inhibits the proliferation of oesophageal cancer cells via up‐regulation of miR‐204

**Figure 5:**
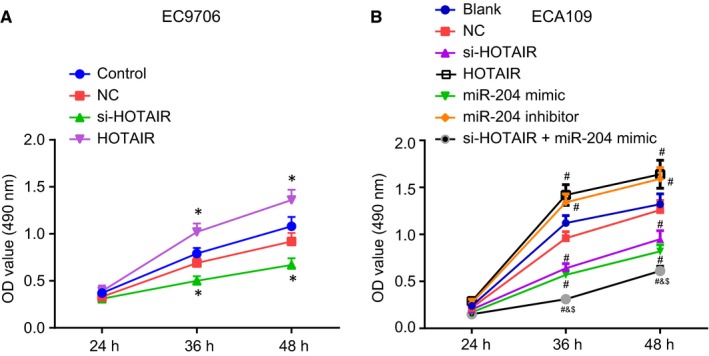
Down‐regulation of HOTAIR inhibits proliferation of oesophageal cancer cells through up‐regulation of miR‐204. (A) the EC9706 cell line treated by si‐HOTAIR shows the lowest OD values from MTT assay. (B) the ECA109 cell line treated with si‐HOTAIR and miR‐204 mimic shows the lowest OD values from MTT assay. *P < 0.05 vs the control group. ^#^ *P* < 0.05 vs the blank group. ^&^ *P* < 0.05 vs the si‐HOTAIR group. ^$^ *P* < 0.05 vs the miR‐204 mimic group. The data are presented as mean ± SD. The values at different time‐points were compared using repeated measurement ANOVA. n = 3. The experiment was independently repeated three times

The functions of HOTAIR and miR‐204 on the viability of oesophageal cancer cells were examined through the application of a MTT assay. The MTT assay (Figure 5A) results revealed there to be no significant difference in the EC9706 cells regarding the cell viability between the control group and NC group (*P* > 0.05), while reduced viability was identified in the si‐HOTAIR group while enhanced levels were found in the HOTAIR group compared to the control group (both *P* < 0.05).

In the ECA109 cell line (Figure 5B), no notable difference was identified regarding the cell viability between the blank and NC groups (*P* > 0.05). When the cell was subjected to different treatments, the cell viability changed. The cell viability was significantly inhibited in the si‐HOTAIR, miR‐204 mimic and si‐HOTAIR + miR‐204 mimic groups while markedly promoted in the miR‐204 inhibitor and HOTAIR groups (all *P* < 0.05). The si‐HOTAIR + miR‐204 mimic group displayed suppressed cell viability when compared to the si‐HOTAIR and miR‐204 mimic groups (both *P* < 0.05). The aforementioned results indicated that down‐regulation of HOTAIR could inhibit the proliferation of oesophageal cancer cells via up‐regulating miR‐204.

### 3.6 Down‐regulation of HOTAIR suppresses migration and invasion of oesophageal cancer cells through up‐regulation of miR‐204

**Figure 6:**
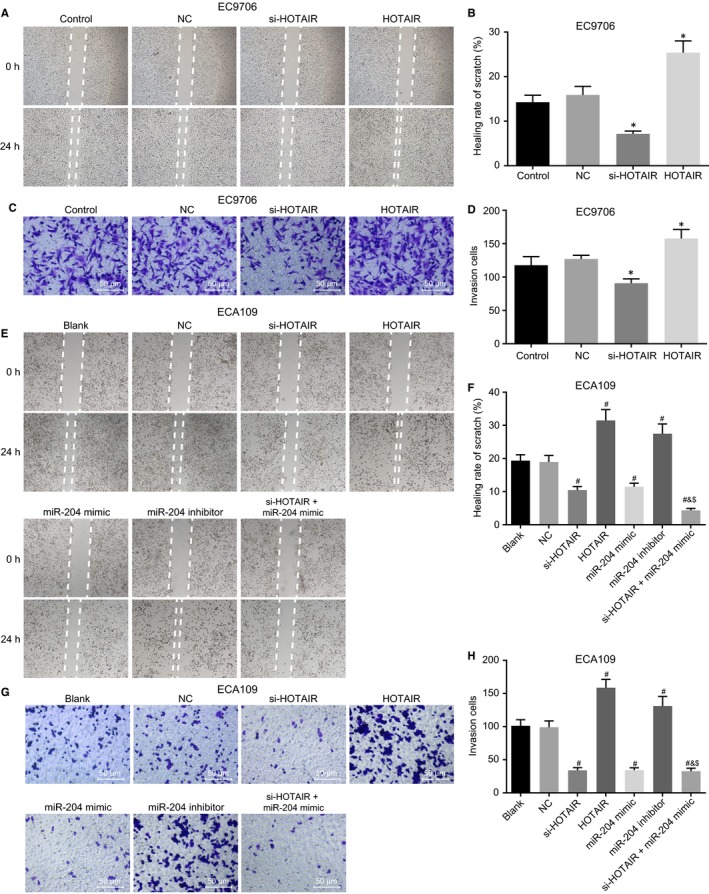
Down‐regulation of HOTAIR or up‐regulation of miR‐204 suppresses migration and invasion of oesophageal cancer cells through up‐regulation of miR‐204. (A,B), the EC9706 cell line treated by si‐HOTAIR shows the lowest migration ability detected by scratch test. (C,D), Transwell assay (×200) reveals that the EC9706 cells treated by si‐HOTAIR shows the lowest invasion ability. (E,F), the ECA109 cells treated by si‐HOTAIR and miR‐204 mimic shows the lowest migration ability detected by scratch test. (G,H), Transwell assay (×200) reveals that the ECA109 cells treated by si‐HOTAIR and miR‐204 mimic shows the lowest invasion ability. *P < 0.05 vs the control group. ^#^ *P* < 0.05 vs the blank group. ^&^ *P* < 0.05 vs the si‐HOTAIR group. ^$^ *P* < 0.05 vs the miR‐204 mimic group. The data are presented as mean ± SD, and analysed by one‐way ANOVA. N = 3. The experiment was independently repeated three times

The effects of HOTAIR and miR‐204 on migration and invasion of oesophageal cancer cells were subsequently investigated by scratch test and Transwell assay, the results of which are shown in Figure 6. In the EC9706 cell line (Figure 6A‐D), no significant difference was identified regarding the scratch healing and invasion abilities of the cells between the control group and NC group (*P* > 0.05). However, in the si‐HOTAIR group, the scratch healing and invasion abilities of the cells were inhibited when compared to the control group, while enhanced levels were observed in the HOTAIR group (all *P* < 0.05).

In the ECA109 cell line (Figure 6E‐H), there was no significant difference detected in relation to the scratch healing and invasion abilities of the cells between the blank and NC groups (*P* > 0.05). In the si‐HOTAIR, miR‐204 mimic and si‐HOTAIR + miR‐204 mimic groups, the scratch healing and invasion abilities of the cells were significantly inhibited whereas they were markedly promoted in the miR‐204 inhibitor and HOTAIR groups when compared to the blank group (all *P* < 0.05). The cells in the si‐HOTAIR + miR‐204 mimic group exhibited a markedly suppressed scratch healing and invasion abilities when compared to the si‐HOTAIR and miR‐204 mimic groups (both *P* < 0.05). These results demonstrate that down‐regulation of HOTAIR can suppress migration and invasion of oesophageal cancer cells via up‐regulating miR‐204.

### 3.7 Down‐regulation of HOTAIR blocks cell cycle progression and induces the apoptosis of oesophageal cancer cells through up‐regulation of miR‐204

**Figure 7:**
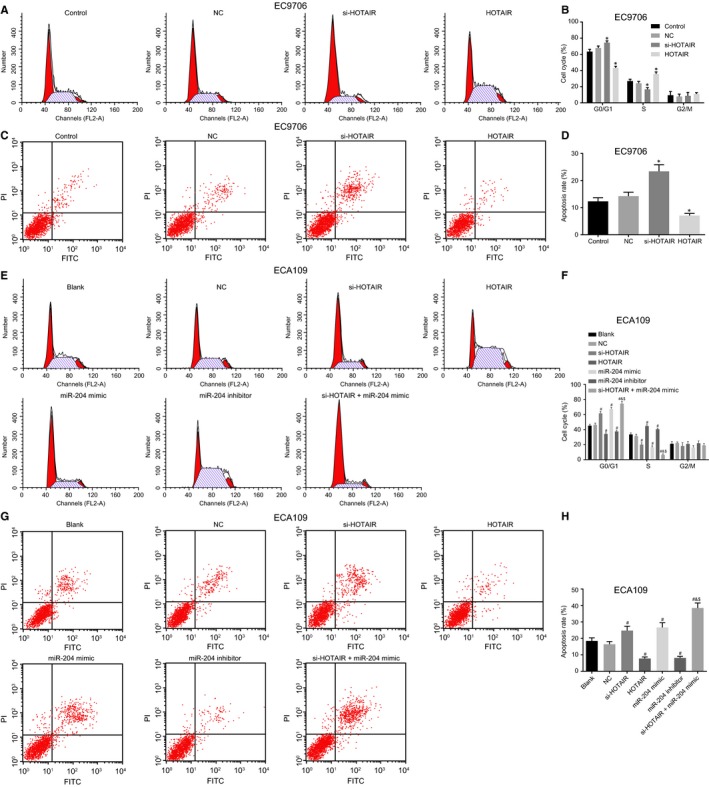
Down‐regulation of HOTAIR accelerates cell cycle progression and induces apoptosis of oesophageal cancer cells through up‐regulation of miR‐204. (A and E), cell cycle distribution of EC9706 and ECA109 cell lines detected by flow cytometry; (B and F) the percentage of PI‐stained cells at the G0/G1, S, and G2/M phases in the EC9706 and ECA109 cell lines; (C and G), oesophageal cancer cells of the EC9706 and ECA109 cell lines in the scatter plots in which the upper left quadrant identifies the necrotic cells (annexin V−/PI+), the upper right quadrant identifies the late apoptotic cells (annexin V+/PI+), the lower left quadrant identifies the live cells (annexin V−/PI−), and the lower right quadrant identifies the early apoptotic cells (annexin V+/PI−). (D and H), the percentage of early and late apoptotic EC9706 and ECA109 cells. *P < 0.05 vs the control group. ^#^ *P* < 0.05 vs the blank group. ^&^ *P* < 0.05 vs the si‐HOTAIR group. ^$^ *P* < 0.05 vs the miR‐204 mimic group. The data are presented as mean ± SD, and analysed by one‐way ANOVA. n = 3. The experiment was independently repeated three times

Flow cytometry was employed in order to ascertain as to whether HOTAIR or miR‐204 could affect the cell cycle distribution and apoptosis of oesophageal cancer cells. Cell cycle distribution after transfection revealed that there was no difference in cell cycle distribution between the control and NC groups (*P* > 0.05) in relation to the EC9706 cell line (Figure 7A‐D), In comparison to the control group, the proportion of cells at the G0/G1 phase remarkably increased in the si‐HOTAIR group while the percentage of cells at the S phase had a significantly higher apoptosis rate (all *P* < 0.05); in the HOTAIR group, with fewer cells arrested at the G0/G1 phase but much more arrested at the S phase along with a reduced apoptosis rate (all *P* < 0.05).

In the ECA109 cell line (Figure 7E‐H), no significant difference was observed in the cell percentage between the blank and NC groups (*P* > 0.05), however, cell proportion at the G0/G1 phase was markedly increased in the si‐HOTAIR, miR‐204 mimic and si‐HOTAIR + miR‐204 mimic groups in comparison to the blank group while the proportion of cells at the S phase was significantly reduced, which was accompanied by a higher apoptosis rate (all *P* < 0.05); the cell proportion at the G0/G1 phase was significantly decreased in the miR‐204 inhibitor and HOTAIR groups, while it was remarkably increased at the S phase, highlighting a lower apoptosis rate (all *P* < 0.05). The si‐HOTAIR + miR‐204 mimic group displayed an elevated apoptosis rate with a higher proportion of cells at the G0/G1 phase and lower cell proportion at the S phase than that in the si‐HOTAIR and miR‐204 mimic groups (all *P* < 0.05). These results provided evidence revealing that the down‐regulation of HOTAIR could mediate cell cycle distribution and induce the apoptosis of oesophageal cancer cells by up‐regulating miR‐204.

### 3.8 Down‐regulation of HOTAIR suppresses oesophageal cancer cell tumourigenicity through up‐regulation of miR‐204

**Figure 8:**
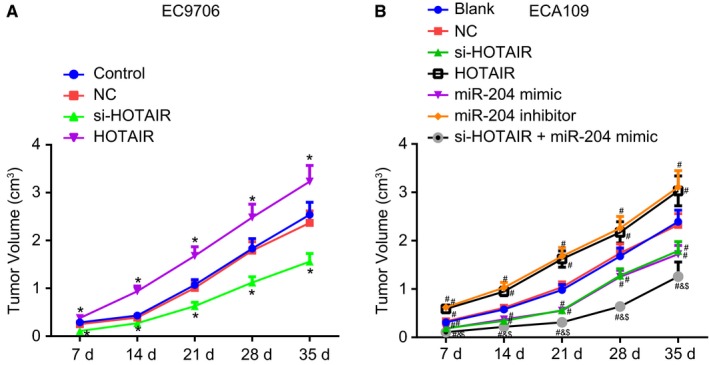
Down‐regulation of HOTAIR suppresses tumour formation through up‐regulation of miR‐204. (A), tumour volume of nude mice after injection of transfected EC9706 cells. (B), tumour volume of nude mice after injection of transfected ECA109 cells. *P < 0.05 vs the control group. ^#^ *P* < 0.05 vs the blank group. ^&^ *P* < 0.05 vs the si‐HOTAIR group. ^$^ *P* < 0.05 vs the miR‐204 mimics group. The data are presented as mean ± SD. The values at different time‐points were compared using repeated measurement ANOVA. N = 6. The experiment was independently repeated three times

Finally, cell tumourigenicity was also assessed in order to elucidate the effects associated with HOTAIR or miR‐204 on oesophageal tumourigenicity via xenograft tumour in nude mice. The results obtained in Figure 8 revealed there to be reduced tumor volume in the si‐HOTAIR group. However, the nude mice in the HOTAIR group exhibited significantly larger tumour size and tumour volume as well as an accelerated tumour growth rate when compared to the control group (all *P* < 0.05), while no significant difference was identified in relation to the tumour size, tumour volume or tumour growth rate between the control and NC groups (both *P* > 0.05).

In the ECA109 cell line, there was no significant difference observed between the blank and NC groups in terms of tumour formation (*P* > 0.05). When this cell line was subjected to different treatment, the size, volume and the growth rate of the tumour in the miR‐204 inhibitor and HOTAIR groups was significantly elevated when compared to the blank group but these tumour formation parameters were all reduced in the si‐HOTAIR, miR‐204 mimic and si‐HOTAIR + miR‐204 mimic groups (all *P* < 0.05). In comparison to the si‐HOTAIR and the miR‐204 mimic groups, the tumour volume reduced significantly in the si‐HOTAIR + miR‐204 mimic group (both *P* < 0.05). Taken together, the results obtained indicate that the down‐regulation of HOTAIR can suppress oesophageal cancer cell tumourigenicity through the up‐regulation of miR‐204.

## 4 DISCUSSION

Oesophageal cancer is a malignancy well‐known for its aggressive nature.^27^ In the current study, we investigated the mechanism by which HOTAIR and miR‐204 influence the development of oesophageal cancer. Our results illustrated that lncRNA HOTAIR has negative impacts on the expression of miR‐204 but positive impacts on the development of oesophageal cancer. Thus, silencing of HOTAIR could suppress the expression of HOXC8 via miR‐204 and inhibit proliferation, migration and invasion while inducing apoptosis of oesophageal cancer cells.

Our results revealed that miR‐204 was poorly expressed in the oesophageal cancer tissues while HOTAIR and HOXC8 were highly expressed. A similar study reported that miR‐204 exhibited lower expression in the neck squamous cell carcinoma tissues in comparison to the healthy adjacent tissues.^28^ Another example showed that miR‐204 suppresses tumour growth through inhibition of light chain 3B (LC3B)‐mediated autophagy in renal clear cell carcinoma.^29^ These evidence illustrated that miR‐204 could perform as a crucial regulator during tumour development. LncRNA HOTAIR and HOXC8 have been highlighted in existing literature as factors involved in tumour formation. HOTAIR has been reported to potentially function as a predictive marker for the metastasis of oesophageal squamous cell carcinoma: with studies linking its elevated expression with poor prognoses.^30^ It has been reported that the up‐regulation of HOXC8 was observed in a variety of cancer types.^31^ Additionally, a previous study asserted that patients with oesophageal squamous cell carcinoma exhibiting high HOXC8 expression levels had shorter median survival time when compared to those with poor levels of HOXC8 expression.^32^

Another key finding of our study revealed that HOTAIR could bind to miR‐204 and miR‐204 could target HOXC8, while we identified that the down‐regulation of HOTAIR resulted in a reduction in the expression of HOXC8 through the up‐regulation of miR‐204. These results suggested that HOTAIR could be a ceRNA of miR‐204, which could inhibit HOXC8. Accumulated reports have demonstrated the role of HOTAIR in cellular processes as a ceRNA: HOTAIR can stimulate the development of glioma, serving as a ceRNA via sponging miR‐126‐5p^33^; HOTAIR promotes to the progression of gastric cancer by acting as a ceRNA of miR‐331‐3p, which is mediating HER2.^34^ Moreover, down‐regulation of HOTAIR decreased the expression of Vimentin and MMP‐9 but increased that of E‐cadherin through up‐regulation of miR‐204. It has been revealed that HOTAIR could increase the expression of MMP‐9 and hence promotes tumour aggressiveness.^35^ Moreover, silencing of HOTAIR has been reported to aid in the up‐regulation of E‐cadherin while reducing the expression of Vimentin.^36^ HOTAIR has been suggested to suppress the expression of E‐cadherin in oral squamous cell carcinoma, thus stimulating tumour cell invasion and metastasis.^37^ In addition, there were studies reporting that miR‐204 could interact with lncRNA UCA1 and target HOXA10.^8^, ^38^ Another example revealed that metazoan miRs, via mRNA cleavage, are capable of suppressing the expression of their natural targets while suggesting their possible involvement in the posttranscriptional restriction of HOX gene expression.^39^ A previous study indicated that miR‐204 could aid in the suppression of epithelial to mesenchymal transition in intrahepatic cholangiocarcinoma cells via targeting slug.^40^ Furthermore, miR‐204 has been reported to aid in elevating the expression of the epithelial marker E‐cadherin while reducing the expression of the mesenchymal marker Vimentin.^40^ Existing literature has illustrated that the overexpression of miR‐204 could contribute to elevated expression of E‐cadherin and reduced expression of N‐cadherin and Vimentin,^41^ a finding of which was consistent with our results. Investigations into cervical cancer cells demonstrated that the overexpression of miR‐204 could reduce the expression of MMP‐9 through the activation of the PI3K/AKT signalling pathway.^42^

Our results further highlighted that the down‐regulation of HOTAIR could inhibit proliferation, invasion and migration, while acting to suppress the apoptosis of oesophageal cancer cell lines and inhibit the tumour formation in nude mice through the up‐regulation of miR‐204. LncRNAs have been widely documented to play a crucial role in the regulation of basic biochemical and cellular activities.^43^ Besides, lncRNA HOTAIR has been demonstrated to possess the ability to stimulate cancer metastasis by reprogramming the chromatin state.^44^ Consistent with the observations of our study, a previous report indicated that the up‐regulation of HOTAIR, with the aids of I‐BET151 treatment, reduced the antiproliferative ability of the BET bromodomain inhibitor.^45^ Additionally, from a breast cancer perspective, the overexpression of HOTAIR has been reported to promote cancer cell proliferation while the down‐regulation of HOTAIR has been found to functionally reduce cancer cell growth as well as cell invasion in prostate cancer.^46^, ^47^ Apart from suppressing HOTAIR, up‐regulation of miR‐204 has been reported to inhibit tumour cell formation. A previous study revealed that miR‐204, by targeting FOXM1, could act to suppress cell invasion in oesophageal cancer,^14^ which was in consistency with the findings of our study. In addition, through the mediation of the SIRT1/p53 signalling pathway, miR‐204 has been reported to promote emitochondrial apoptosis in doxorubicin‐treated prostate cancer cells.^48^

**Figure 9:**
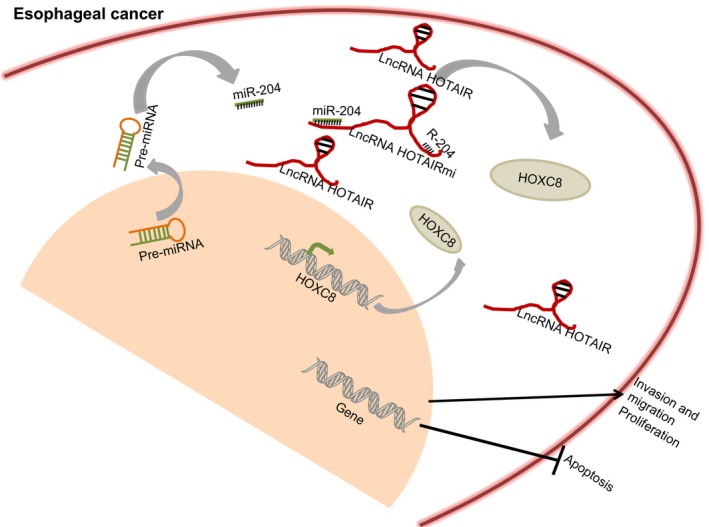
The mechanism diagram depicting that lncRNA HOTAIR functions as a ceRNA of miR‐204 to increase the expression of HOXC8, and thus enhancing progression of oesophageal cancer

To conclude, this study illustrated that lncRNA HOTAIR could function as a ceRNA of miR‐204, and the silencing of HOTAIR could reduce expression of HOXC8, which ultimately inhibited the proliferation, migration and invasion of oesophageal cancer cells (Figure 9). This suggested that repression of HOTAIR could be clinically helpful to suppress oesophageal cancer progression and HOTAIR could be a promising target for oesophageal cancer treatment. However, an additional molecular mechanism by which HOTAIR works has been reported involving mediation on epithelial genes expression (ie E‐cadherin) through the recruitment of PRC2.^49^, ^50^ Therefore, more researches on mechanisms of HOTAIR‐based oesophageal cancer therapeutics are required to explore the application potential.

## AUTHORS CONTRIBUTIONS

AHW and PT designed the study. YZ and ZBY collated the data, carried out data analyses and produced the initial draft of the manuscript. XTZ and LNL contributed to drafting and polishing the manuscript. All authors have read and approved the final submitted manuscript.

## COMPETING INTERESTS

The authors declare that they have no competing interests.
